# Bacterial Community Selection of *Russula griseocarnosa* Mycosphere Soil

**DOI:** 10.3389/fmicb.2020.00347

**Published:** 2020-03-25

**Authors:** Fei Yu, Jun-Feng Liang, Jie Song, Sheng-Kun Wang, Jun-Kun Lu

**Affiliations:** Key Laboratory of State Forestry Administration on Tropical Forestry Research, Research Institute of Tropical Forestry, Chinese Academy of Forestry, Guangzhou, China

**Keywords:** *Russula griseocarnosa*, mycosphere, functional diversity, Miseq sequencing, soil bacterial community

## Abstract

*Russula griseocarnosa* is a wild, ectomycorrhizal, edible, and medicinal fungus with high economic value in southern China. *R. griseocarnosa* fruiting bodies cannot be artificially cultivated. To better understand the effects of abiotic and biotic factors on *R. griseocarnosa* growth, the physicochemical properties of *R. griseocarnosa* and its associated bacterial communities were investigated in two soil types (mycosphere and bulk soil) from Fujian, Guangdong, and Guangxi Provinces. The results revealed that the diversity, community structure, and functional characteristics of the dominant mycosphere bacteria in all geographical locations were similar. Soil pH and available nitrogen (AN) are the major factors influencing the mycosphere–soil bacterial communities’ structure. The diversity of soil bacteria is decreased in *R. griseocarnosa* mycosphere when compared with the bulk soil. *Burkholderia-Paraburkholderia*, *Mycobacterium*, *Roseiarcus*, *Sorangium*, *Acidobacterium*, and *Singulisphaera* may also be mycorrhiza helper bacteria (MHB) of *R. griseocarnosa*. The functional traits related to the two-component system, bacterial secretion system, tyrosine metabolism, biosynthesis of unsaturated fatty acids, and metabolism of cofactors and vitamins were more abundant in *R. griseocarnosa* mycosphere soil. The mycosphere soil bacteria of *R. griseocarnosa* play a key role in *R. griseocarnosa* growth. Application of management strategies, such as N fertilizer and microbial fertilizer containing MHB, may promote the conservation, propagation promotion, and sustainable utilization of *R. griseocarnosa*.

## Introduction

Soil is a dynamic biological matrix and an essential part of the terrestrial ecosystem. Soil microbes can participate in crucial processes such as biogeochemical cycles and play a role in different environmental conditions (Cao et al., 2016). Soil bacteria play an influential role in the nitrogen cycle, such as N fixation (Lladó et al., 2017), which is associated with the richness of ectomycorrhizal fungi (Allison et al., 2007; Mediavilla et al., 2019). Soil bacteria, especially mycorrhiza helper bacteria (MHB), can improve the ability of plant roots to form mycorrhiza (Aspray et al., 2006), promote the growth of fungi on soil or root surface, and trigger the germination of fungi in soil (Frey-Klett et al., 2007, 2011). Bacteria may have a variety of symbiotic functions in mushrooms, including inhibiting pathogens and antagonists, improving spore distribution, provisioning of the growth regulators and vitamins (Riedlinger et al., 2006), and increasing mushroom production (Pent et al., 2017). Bacteria were found in fungal hyphae, mycorrhiza, and fungal fruit bodies (Boer et al., 2005; Pent et al., 2017). These MHB serve as biofertilizers to promote fruiting bodies’ formation and increase their productivity (Young et al., 2013). Ectomycorrhizal fungi release many hyphae that contribute to the absorption of water and nutrients (Martin et al., 2007) and can also be used as carriers to transport bacteria (Boer et al., 2005).

*Russula griseocarnosa* is a wild, edible, medicinal, and ectomycorrhizal symbiont fungi distributed broadly in southern China (Yu et al., 2020). The fruiting bodies of *R. griseocarnosa* cannot be artificially cultivated (Chen et al., 2010; Ming et al., 2014). *R. griseocarnosa* has high economic value; its flesh has high nutritional value (Chen et al., 2010; Ming et al., 2014). *R. griseocarnosa* has been proved to have beneficial effects on dispelling or preventing heart disease and softening brain veins (Chen et al., 2010) when used as a functional food (Chen et al., 2010). *R. griseocarnosa* polysaccharides have antioxidant activities (Ming et al., 2014) and inhibit the proliferation of cervical cancer cells (Yuan et al., 2017; Liu et al., 2018). Based on the location and the quality of *R. griseocarnosa*, the fruiting bodies of *R. griseocarnosa* can sell for $35–$45/kg, while dried of *R. griseocarnosa* are sold for $140–$180/kg (Ming et al., 2014), with prices increasing. *R. griseocarnosa* hyphae aggregate densely with the soil around ectomycorrhizal host trees such as *Betula platyphylla*, *Castanopsis carlesii*, *Pinus massoniana*, and *Psychotria asiatica*. In the symbiotic relationship between fungi and host trees, the fungus can absorb essential elements, especially phosphorus (Hall et al., 2003), to promote the growth of trees, and the trees can provide carbohydrates to the fungus (Giomaro et al., 2005). The fruit body formation of ectomycorrhizal mushrooms must have a symbiotic relationship with plants under certain conditions, and the process is hard to achieve artificially for most of the edible ectomycorrhizal fungi (Hall et al., 2003; Giomaro et al., 2005), such as *R. griseocarnosa*. There is evidence that several bacteria are selected in the mycosphere of the ectomycorrhizal *Laccaria proxima* (Warmink et al., 2009). *Pseudomonas* and *Burkholderia* are the main bacterial communities in the fruit bodies and in the soil environment of *Russula decolorans* (Pent et al., 2017). The *Pseudomonas* communities are significantly increased in the *L. proxima* mycospheres compared with the corresponding bulk soil (Warmink et al., 2009). Further evidence reveals that bacteria can trigger (Noble et al., 2009) or inhibit (Munsch et al., 2002; Yun et al., 2013) fruiting bodies’ formation of mushrooms. The composition of bacteria within fruiting bodies can be affected directly or indirectly by soil bacterial communities (Antony-Babu et al., 2013), suggesting that *R. griseocarnosa* may also have helper bacteria to grow and maintain mycelium in the soil.

Soil physicochemical properties, fungi, and other factors may affect the community structure of soil microbial communities (Garbeva et al., 2004). Singh et al. (2008) showed that plant species affect rhizosphere fungi but not rhizosphere bacteria. Soil microbial community and related environmental parameters drive rhizosphere bacterial community structure more than plant genotypes or species (Bulgarelli et al., 2012; Vandenkoornhuyse et al., 2015). The soil contains a variety of bacterial communities shaped by environmental forces (Rousk et al., 2010). These environmental forces may indirectly affect the structure of the bacterial communities in the mycelium and the fruiting bodies of fungi (Warmink et al., 2009; Pent et al., 2017). The effects of bacteria on ectomycorrhizal fungi can vary according to soil factors such as pH and carbon availability (Brulé et al., 2001; Pent et al., 2017; Oh and Lim, 2018). The bacteria in the surrounding soil are filtered by the conditions created by the fruiting bodies, and some bacteria are still retained in the fruiting bodies (Antony-Babu et al., 2013; Pent et al., 2017). MHB are not plant-specific but selective for fungal species (Pivato et al., 2009). This selectivity has been found in fungi that select the soil bacterial communities based on fungal (Halsey et al., 2016) and specific soil properties, such as pH and soil organic carbon (SOC) content (Pent et al., 2017). The non-random selection may depend on their symbiotic functions or habitat requirements (Pent et al., 2017). This selectivity is more conducive to the development of fungal fruiting bodies. Fruiting body formation of *L. proxima* can be triggered by *Pseudomonas* communities (Warmink et al., 2009). Bacterial metabolites, nutrients, or stimuli can have a positive or negative effect on fungal growth or spore germination (Oh and Lim, 2018). Leyval and Berthelin (1991) speculated that bacteria could dissolve soil nutrients and cooperate with ectomycorrhizal fungi to increase the diffusion of host roots.

We aimed to explore the characteristics of soil bacteria related to the growth of *R. griseocarnosa* by comparing the diversity, community structure, and functional profiles of bacteria in the mycosphere and bulk soil. We used Miseq sequencing to expand the research scope and improve the accuracy by comparing soil types in different geographical locations. Also, PICRUSt was used to predict and compare the functional spectrum of bacteria in the mycosphere soil of *R. griseocarnosa*. We expect this study will not only help us to understand the interaction between *R. griseocarnosa* and soil bacteria but also provide a theoretical basis for the conservation and propagation of *R. griseocarnosa*.

## Materials and Methods

### Sample Collection

Eighty soil samples from 10 *R. griseocarnosa* growth sites were collected. Growth sites were distributed in three provinces of China within the longitudinal ranges from 110°38′ to 117°35′ during July 2017 (Table 1). The environment of each site is composed of forest lands with different and distinct vegetation (Table 1). All regions encompass altitude ranges from 38 to 708 m above sea level and a fruiting air temperature range from 21 to 38°C. Geographic distance range from 6.50 to 763.48 km (Supplementary Table S1).

**TABLE 1:** Site information used for this study.

Sample	Location	Replicate	Vegetation	Longitude (E)	Latitude (N)	Altitude (m)	pH	SOC (g/kg)	AN (mg/kg)	AP (mg/kg)	AK (mg/kg)
DT	Datian Co., Fujian Prov.	5	*Castanopsis carlesii*	117°35′44.70″	25°49′20.17″	708	4.09	77.28	394.13	6.52	160.76
DTCK	Datian Co., Fujian Prov.	3	*Dendropanax dentigerus*	117°35′44.70″	25°49′20.17″	708	4.09	69.42	321.54	4.19	121.84
YA	Yongan Co., Fujian Prov.	5	*Schima superba*	117°21′54.19″	25°56′30.97″	183	4.14	58.26	344.17	3.73	152.03
YACK	Yongan Co., Fujian Prov.	3	*Dalbergia hancei*	117°21′54.19″	25°56′30.97″	183	3.97	89.19	312.34	2.24	129.93
ZP	Zhangping Co., Fujian Prov.	5	*Toona ciliata*	117°25′11.99″	25°17′24.66″	168	4.20	71.74	369.88	19.60	318.03
ZPCK	Zhangping Co., Fujian Prov.	3	*Toona ciliata*	117°25′11.99″	25°17′24.66″	168	3.88	139.07	230.17	6.31	154.93
FS	Fengshun Co., Guangdong Prov.	5	*Choerospondias axillaris*	116°16′57.73″	24°5′25.06″	147	4.39	25.79	214.18	4.80	231.93
FSCK	Fengshun Co., Guangdong Prov.	3	*Choerospondias axillaris*	116°16′57.73″	24°5′25.06″	147	4.46	12.88	132.48	2.44	112.14
JL	Jiaoling Co., Guangdong Prov.	5	*Castanopsis chinensis*	116°13′55.14″	24°35′14.50″	338	3.99	99.36	643.08	10.15	241.31
JLCK	Jiaoling Co., Guangdong Prov.	3	*Castanopsis chinensis*	116°13′55.14″	24°35′14.50″	338	6.43	16.78	102.12	80.04	79.25
HTC	Huangtianchong, Guangxi Prov.	5	*Castanopsis chinensis*	110°41′59.24″	23°10′33.92″	149	4.55	60.39	251.71	4.98	95.75
HTCCK	Huangtianchong, Guangxi Prov.	3	*Castanopsis chinensis*	110°41′59.24″	23°10′33.92″	149	4.50	25.63	179.40	1.67	71.70
JJ	Jinji Town, Guangxi Prov.	5	*Psychotria asiatica*	110°49′18.61″	23°13′36.54″	38	4.17	43.07	224.94	9.40	153.32
JJCK	Jinji Town, Guangxi Prov.	3	*Psychotria asiatica*	110°49′18.61″	23°13′36.54″	38	4.30	46.27	172.50	3.65	89.49
LJ	Lingjing Town, Guangxi Prov.	5	*Camellia reticulata*	110°38′52.25″	23°8′32.83″	69	4.30	36.73	231.01	3.77	130.68
LJCK	Lingjing Town, Guangxi Prov.	3	*Camellia reticulata*	110°38′52.25″	23°8′32.83″	69	4.65	14.98	133.40	5.30	181.14
THL	Tianhongling, Guangxi Prov.	5	*Psychotria asiatica*	111°15′48.89″	23°41′47.33″	328	4.21	40.37	321.82	4.10	111.60
THLCK	Tianhongling, Guangxi Prov.	3	*Psychotria asiatica*	111°15′48.89″	23°41′47.33″	328	4.05	34.40	252.54	3.26	85.72
YY	Youyi, Cangwu Co., Guangxi Prov.	5	*Ardisia quinquegona*	111°33′35.09″	23°41′30.84″	43	4.11	34.45	275.45	5.19	295.65
YYCK	Youyi, Cangwu Co., Guangxi Prov.	3	*Ardisia quinquegona*	111°33′35.09″	23°41′30.84″	43	4.22	26.08	246.56	3.63	123.46

*SOC, AN, AP, and AK represent soil organic carbon, available nitrogen, available phosphorus, and available potassium, respectively.*

The geographic location and vegetative characteristics are listed in Table 1. At each site, the five *R. griseocarnosa* fruiting bodies were excavated at a depth of 10 cm using a sterile hand trowel; mycosphere soil was then transferred into a sterile polythene bag (Warmink and van Elsas, 2008; Oh et al., 2016). Samples were collected in the no-fruiting-bodies area with a lateral distance of 40 cm from the *R. griseocarnosa* and will herein be referred to as “bulk soil” (Warmink and van Elsas, 2008). One fraction of the samples was frozen using liquid nitrogen and stored at −70°C for DNA extraction. The remaining fraction was air-dried and sieved using a 2 mm mesh and then used for physicochemical analysis.

Air-dried samples were used to determine soil pH using a 2 mm mesh with a 1:2.5 (w/v) soil-to-water ratio suspension (Wu et al., 2000). SOC was measured by dichromate oxidation (Nelson and Sommers, 1996). Available phosphorus (AP) was measured using the sodium hydrogen carbonate solution-Mo-Sb anti spectrophotometric (Retamal-Salgado et al., 2017). Soil available potassium (AK) was measured by flame photometry (Zhao et al., 2014). Available nitrogen (AN) was determined by potassium persulfate oxidation (Liu et al., 2015).

### DNA Isolation and PCR Amplification

Soil DNA was extracted from 0.30 g soil using the Ezup Column Soil DNA kit (Sangon Biotech, Shanghai) according to the manufacturer instructions (Griffiths et al., 2000). Samples were placed into 1.5 ml centrifuge tubes with 500 mg of glass beads. 400 μl of Buffer SCL at 65°C was added to the samples, followed by incubation at 65°C in a water bath for 5 min. Samples were then centrifuged for 3 min, and the supernatant was collected. An equal volume Buffer SP was added to the supernatant and incubated on ice for 10 min. Following incubation, 200 μl of β-Mercaptoethanol was added, and samples were further centrifuged for 3 min. The supernatant was collected, and 1.5 volumes of Buffer SB were added. Samples were washed twice with 700 and 300 μl Wash Solution, respectively. Finally, 80 μl TE Buffer was added to the center of the adsorption membrane, and the DNA solution was obtained by centrifugation at 12,000 rpm for 3 min. DNA concentration and purity were measured by NanoDrop 2000 spectrophotometer (Thermo Scientific, Wilmington, DE, United States).

The V3-V4 regions of bacterial 16S were amplified by primers 338F (5′-ACTCCTACGGGAGGCAGCAG-3′) and 806R (5′-GGACTACHVGGGTWTCTAAT-3′) (Mori et al., 2014). The PCR reactions were conducted using the following program: 95°C for 3 min, followed by 35 cycles of 95°C for 30 s, 55°C for 30 s, 72°C for 45 s, and a final extension of 72°C for 10 min in a GeneAmp 9700 thermocycler PCR system. PCR reactions were performed as follows: 4 μl 5 × FastPfu buffer, 2 μl 2.5 mM dNTPs, 0.8 μl of each primer (5 μM), 0.4 μl FastPfu polymerase, 0.2 μl 2.0 g/l BSA, 2 μl 50 mg/l template DNA, and 9.8 μl ddH_2_O in a 20 μl total volume. All PCR products were collected from 2% agarose gels and purified using a DNA gel extraction kit (Axygen Biosciences, Inc., United States) and quantified before sequencing.

### Miseq Sequencing

Purified products were assembled in an equal volume and sequenced (2 × 300 bp) using Illumina’s Miseq platform in Majorbio Bio-Pharm Technology Co., Ltd., Shanghai, China. The raw reads were deposited into the NCBI Sequence Read Archive (SRA) database (Accession Number: PRJNA553654).

### Bioinformatic Analysis of the 16SrRNA Amplicons

Raw fastq were demultiplexed, quality-filtered, and merged using the following standards: (1) truncate the 300 bp reads where the average quality score <20 over a 50 bp; the truncated read codes less than 50 bp were discarded; (2) precise barcode matching sequences were included, and two nucleotide mismatch in primer matching or reads containing ambiguous characters were deleted; (3) only assemble overlapped sequences exceeding 10 bp according to overlapped sequences; and (4) unassembled readings were discarded.

Operational taxonomic units (OTUs) were clustered at 97% similarity cutoff value, and chimeric sequences were identified and removed using USEARCH^1^ (version 7.0). The 16S rRNA gene sequence was analyzed by SILVA (SSU123) database using a confidence threshold of 70% (Cole et al., 2013; Quast et al., 2013). The subsampling was based on the minimum sample sequence with equal sequencing depth (16,175 sequences per followed by clustering) (Ye et al., 2017). Diversity metrics, that is, richness (observed species), Chao richness index, Shannon diversity index, and coverage and phylogenetic diversity were calculated based on OTU tables using mother (v.1.30.1). The indexes describe the structure of bacterial communities.

### Statistical Analysis

The statistical analysis was conducted using the online platform of Majorbio I-Sanger Cloud Platform^2^. The results of the two groups of data were consistent with the normal distribution, and the variance of the two groups was not equal. Therefore, the results were expressed as mean values and two-group statistical analyses using Welch’s *t*-test (Delacre et al., 2017). The bar of diversity index represents the mean ± standard error. Significant correlations are expressed as: ^∗^ 0.01 < *p* ≤ 0.05; ^∗∗^ 0.001 < *p* ≤ 0.01; ^∗∗∗^*p* ≤ 0.001.

LEfSe was used to identify taxa that differed consistently using the default parameters (LDA Score >2, *p* < 0.05). LEfSe was applied in the identification of mycosphere and bulk soil biomarkers of microbiomes at the genus levels. The biomarkers were classified according to their statistical significance. The results were visualized by using bar charts and cladograms (Segata et al., 2011).

Mantel Tests (1967) with 999 permutations were used to test the Bray–Curtis correlation between soil/site properties and bacterial community structure by QIIME (Caporaso et al., 2010). ANOSIM analysis of the relationship of sites was performed using R’s Vegan package (version 3.3.3) (Oksanen et al., 2017). To analyze the relationship between taxa and the soil/site properties, variation portioning analysis (VPA) was done using R’s Vegan package (Oksanen et al., 2017). The OTUs and soil/site properties were used in the analysis. Detrended correspondence analysis (DCA) was done based on OTUs. Principle component analysis (PCA) plot was drawn by R’s Vegan package (Oksanen et al., 2017).

Spearman’s correlation coefficients among the top 30 mycosphere’s bacterial genera and soil properties were calculated and displayed as a heat map using R’s pheatmap package (Kolde, 2019). The Spearman’s correlation analysis of soil properties and the diversity indexes were calculated by SPSS21.0.

### 16S rRNA Functional Predictions

The microbial function was predicted by PICRUSt (Langille et al., 2013; Oh et al., 2016). OTUs was assigned with QIIME’s command “pick_closed_otus” with 97% similarity in Greengenes13.5 database. Then, the predicted functions were blasted to the KEGG (Kyoto Encyclopedia of Genes and Genomes) database, and statistical differences among groups were compared by STAMP software (Parks and Beiko, 2010). Welch’s *t*-test and Storey False Discovery Rate (FDR, *p* < 0.05) were performed for two groups (Storey, 2007).

## Results

### Site Sampling of Mycosphere and Bulk Soil

Soil organic carbon at the collection sites ranged from 12.88 to 139.07 g/kg (Table 1). Soil pH was between 3.88 and 6.43 at the collection sites. The soil contents of available nitrogen (AN, 102.12–643.08 mg/kg), available phosphorus (AP, 1.67–80.04 mg/kg), and available potassium (AK, 71.70–318.03 mg/kg) showed rich changes in collection sites (Table 1). The geographical distance ranges from 6.50 to 763.48 km (Supplementary Table S1).

Of all sites, the soil pH of YA (*p* = 0.014) and ZP (*p* = 0.001) was significantly higher in the mycosphere soil, while soil pH of LJ (*p* < 0.001), JL (*p* < 0.001) and JJ (*p* = 0.032) were significantly lower in the mycosphere soil. The SOC of LJ (*p* = 0.016), HTC (*p* = 0.022), and JL (*p* = 0.010) was significantly higher in the mycosphere soil, while the SOC of YA (*p* = 0.027) and ZP (*p* = 0.001) was significantly lower in the mycosphere soil. The AN of LJ (*p* = 0.041), DT (*p* = 0.006), JL (*p* = 0.018), and ZP (*p* < 0.001) was significantly higher in the mycosphere soil. The AP of YA (*p* = 0.004) and ZP (*p* = 0.044) was significantly higher in the mycosphere soil. The AK of JJ (*p* = 0.020), YA (*p* = 0.028), and ZP (*p* < 0.001) was significantly higher in the mycosphere soil. In most sites with mycorrhiza soil, the content of AN, AK, and AP was significantly higher than those of bulk soil. The results showed that mycosphere soils were more nutrient-rich compared with bulk soils (Supplementary Table S2).

### Bacteria Communities and Structure in Mycosphere and Bulk Soil

#### Diversity of Bacterial Community

**FIGURE 1:**
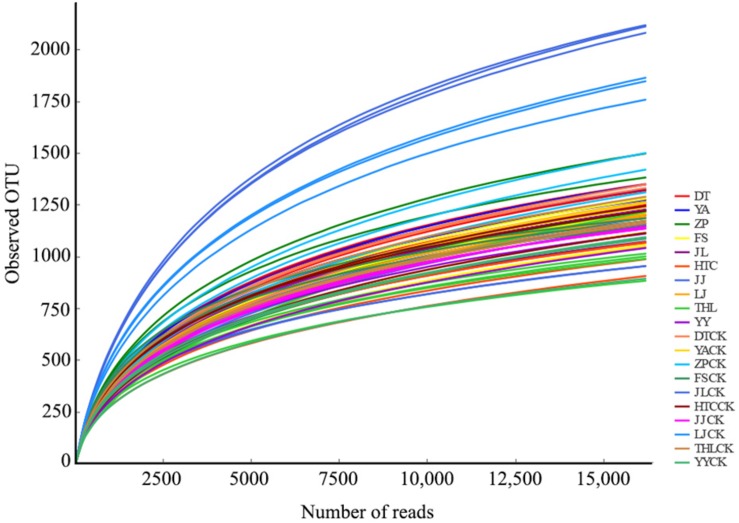
Rarefaction curves of bacterial OTUs.

**FIGURE 2:**
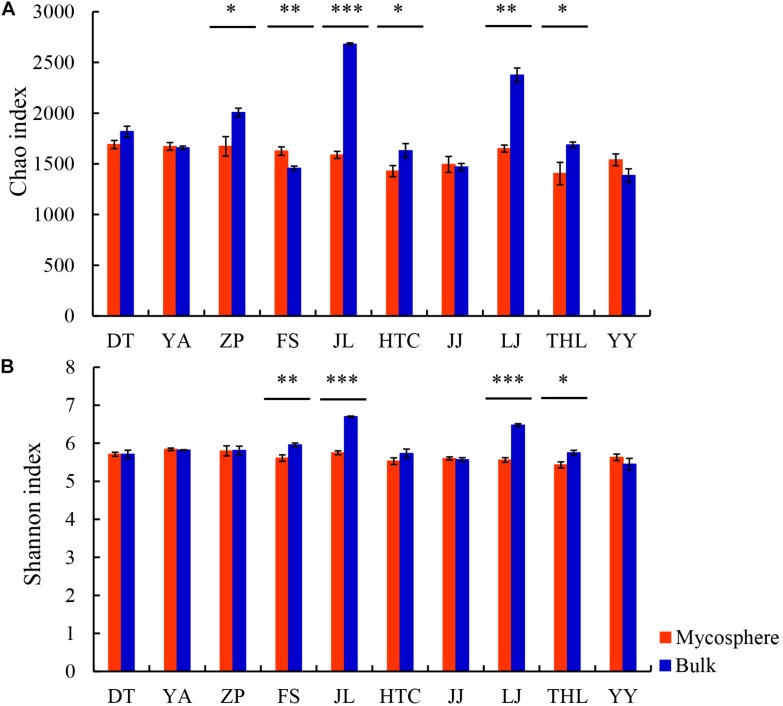
Comparison of Chao **(A)** and Shannon **(B)** indexes between mycosphere and bulk soil. Significant differences by *p < 0.05; **p < 0.01 and ***p < 0.001.

Each sample had 16,175 bacterial sequences for further analysis (Figure 1). A total of 6,014 OTUs were delineated at a 97% similarity level. We investigated the distinctiveness between mycosphere and bulk bacterial communities with samples from ten different sites. Chao and Shannon indexes of mycosphere samples from JL, LJ, and THL were significantly lower than in bulk soil (Figure 2). The Chao index of HTC site (*p* = 0.010) and ZP site (*p* = 0.010) was significantly lower than that of bulk soil, while the Shannon index showed no significant difference in bulk soil. Only four sites reported no significant difference between the Chao and Shannon indexes in regard to mycosphere and bulk soil. The bacterial community structure clustered significantly with soil compartments in ten sites (ANOSIM; bacteria: *R* = 0.74, *p* = 0.001).

#### Keystone Species in Mycosphere and Bulk Soils

**FIGURE 3:**
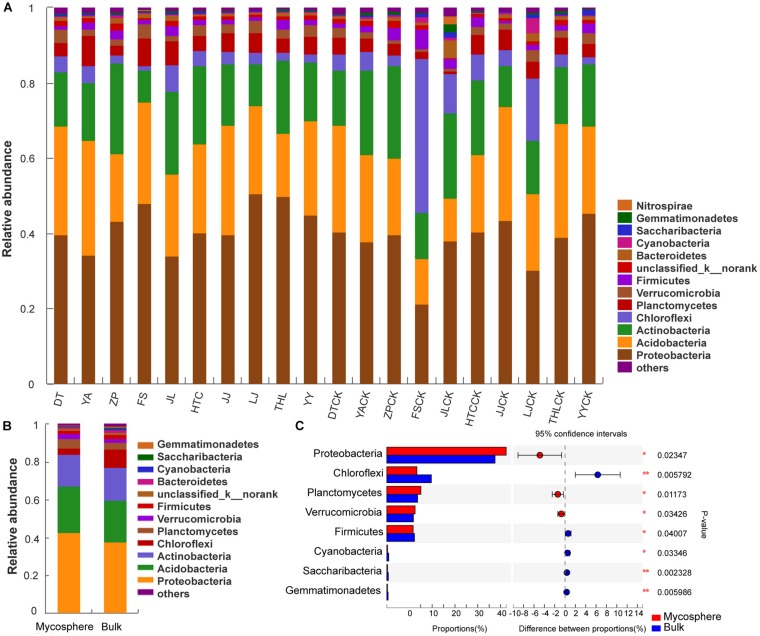
Comparison of phyla between mycosphere and bulk soil. **(A)** The abundances of phyla of each site. **(B)** Comparison of the average abundance of phylum in mycosphere and bulk soil. **(C)** Significant differences among the abundances of phyla between mycosphere and bulk soil. Significant differences by *p < 0.05 and **p < 0.01.

There was a total of 6,014 bacterial OTUs obtained from the ten sites, clustered into 38 phyla. *Proteobacteria*, *Acidobacteria*, *Actinobacteria*, and *Chloroflexi* were the dominant phyla present in soil samples (Figure 3A), accounting for 86.99 and 86.53% of the total species in mycosphere and bulk soil samples, respectively (Figure 3B). *Cyanobacteria*, *Saccharibacteria*, *Gemmatimonadetes*, and *Nitrospirae* phyla were also present in all samples examined but at a lower species richness. *Proteobacteria* (*p* = 0.023), *Planctomycetes* (*p* = 0.012), and *Verrucomicrobia* (*p* = 0.034) were significantly higher in mycosphere soil, while *Chloroflexi* (*p* = 0.006), *Firmicutes* (*p* = 0.040), *Cyanobacteria* (*p* = 0.033), *Saccharibacteria* (*p* = 0.002), and *Gemmatimonadetes* (*p* = 0.006) were significantly lower in mycosphere soil (Figure 3C).

At the phylum level, the relative abundances of *Acidobacteria* (*p* = 0.022) and *Planctomycetes* (*p* = 0.016) were significantly enriched in YA mycosphere soil, while *Actinobacteria* (*p* = 0.015), *Saccharibacteria* (*p* = 0.013), and *Gemmatimonadetes* (*p* = 0.030) were significantly higher in the YA bulk soil (Supplementary Table S3). The relative abundances of *Proteobacteria* (*p* = 0.004), *Acidobacteria* (*p* = 0.005), *Planctomycetes* (*p* = 0.030), and *Verrucomicrobia* (*p* = 0.017) were significantly higher in FS mycosphere soil, while *Chloroflexi* (*p* < 0.001), *Actinobacteria* (*p* = 0.011), *Firmicutes* (*p* = 0.008), and *Cyanobacteria* (*p* < 0.001) were significantly higher in FS bulk soil (Supplementary Table S3). At the phylum level, the relative abundances of *Acidobacteria* (*p* = 0.015) and *Planctomycetes* (*p* = 0.019) were significantly higher in JL mycosphere soil, while *Bacteroidetes* (*p* = 0.013), *Saccharibacteria* (*p* = 0.022), *Gemmatimonadetes* (*p* < 0.001), and *Nitrospirae* (*p* = 0.012) were significantly lower (Supplementary Table S3). The relative abundances of *Proteobacteria* (*p* = 0.006) were significantly higher in LJ mycosphere soil, while *Chloroflexi* (*p* < 0.001), *Cyanobacteria* (*p* = 0.017), and *Bacteroidetes* (*p* = 0.018) were significantly higher in LJ bulk soil (Supplementary Table S3). The relative abundances of *Gemmatimonadetes* (*p* = 0.001) were significantly higher in the HTC bulk soil (Supplementary Table S3). The relative abundances of *Acidobacteria* (*p* = 0.046) were significantly higher in the THL bulk soil. These results show that *Proteobacteria*, *Acidobacteria*, *Planctomycetes*, and *Verrucomicrobia* were significant higher in mycosphere soil, which was consistent with the overall analysis (Supplementary Table S3).

**FIGURE 4:**
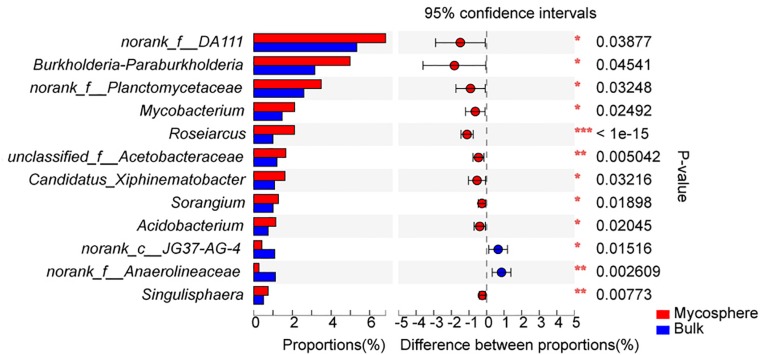
Significant differences among the top 30 genera between mycosphere and bulk soil. Significant differences by *p < 0.05; **p < 0.01 and ***p < 0.001.

**FIGURE 5:**
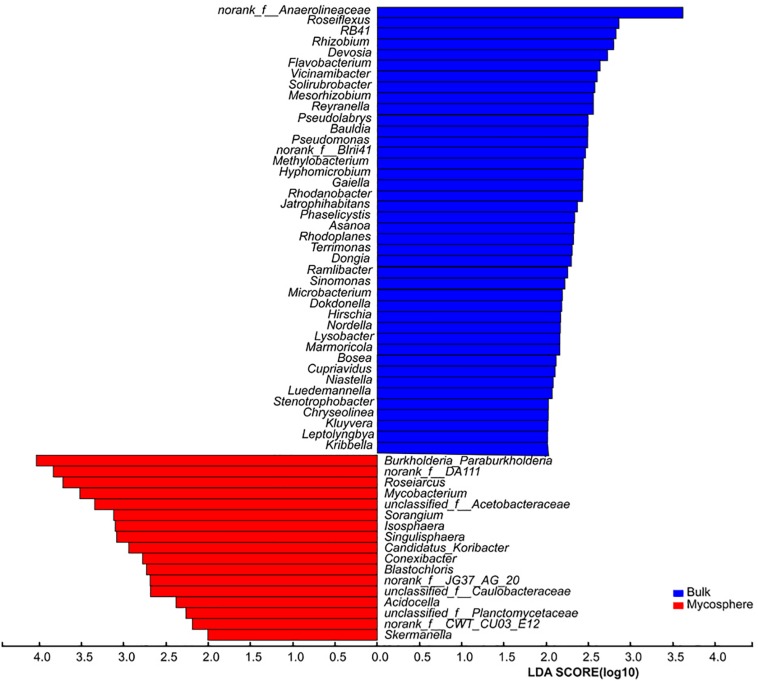
LDA scores showed all the significant genus differences between mycosphere and bulk soil.

Over 700 genera were found in the sequencing data. The relative abundance of 92 bacterial genera was over 1%. In top 30 genera, the norank_f__DA111 (*p* = 0.039), *Burkholderia-Paraburkholderia* (*p* = 0.045), *Mycobacterium* (*p* = 0.025), *Roseiarcus* (*p* < 0.001), *Candidatus_Xiphinematobacter* (*p* = 0.032), *Sorangium* (*p* = 0.019), *Acidobacterium* (*p* = 0.020), and *Singulisphaera* (*p* = 0.008) were significantly higher in mycosphere soil samples (Figure 4 and Supplementary Table S4), while the norank_c__JG37-AG-4 (*p* = 0.015) and norank_f__Anaerolineaceae (*p* = 0.003) were significantly higher in bulk soil (Figure 4). For all genera, mycosphere and bulk soil groups were represented by cladograms, and the LDA scores of two were proved by LEfSe (Figure 5).

#### Abiotic and Biotic Factors in *R. griseocarnosa* Mycosphere and Bulk Soils

Soil pH, SOC, AN, AP, and AK produce the highest variability in bacterial community structures for both mycosphere and bulk soil, as demonstrated by the Mantel test (Table 2). To quantify the effects of the soil properties and the altitude on mycosphere bacterial communities, a variance partitioning analysis (VPA) was performed. A matrix of the soil properties’ relationship with the soil bacterial community was constructed using RDA analysis.

**TABLE 2:** The Mantel test analysis in soil properties.

Group	pH	SOC	AN	AP	AK	Altitude
Mycospher	0.238 (0.009)	0.183 (0.025)	0.0231 (0.019)	0.215 (0.013)	0.137 (0.043)	0.0915 (0.134)
Bulk	0.754 (0.001)	0.384 (0.001)	0.523 (0.002)	0.518 (0.001)	0.091 (0.301)	0.0767 (0.397)

*SOC, AN, AP, and AK represent soil organic carbon, available nitrogen, available phosphorus, and available potassium, respectively.*

**FIGURE 6:**
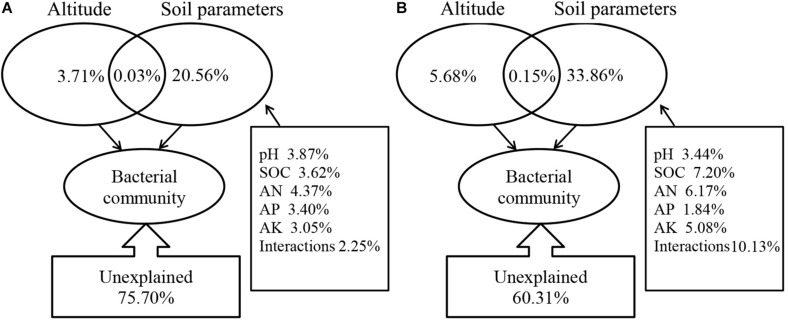
Variation partition analysis (VPA) of soil/site properties on the bacterial community. **(A)** Mycosphere soil. **(B)** Bulk soil.

Correlation analysis showed that there was a significant correlation between the soil parameters and the soil bacterial community structure. These variables explain the changes in bacterial community structure in the mycosphere (24.30%) and bulk soil (39.69%) (Figure 6). Soil parameters constituted 20.56%, altitude constituted 3.71%, and interactions between the soil parameters and altitude explained 0.03% of the variations in the mycosphere bacterial communities (Figure 6A). Meanwhile, for bulk soil, soil parameters explained 33.86%, altitude explained 5.68%, and interactions between the soil parameters and altitude explained the 0.15% of the variations in bacterial communities (Figure 6B). The soil pH and AN were identified as the main contributing factors to the soil parameter and explained the bacterial communities’ variety in the mycosphere at 3.87 and 4.37%, respectively (Figure 6).

**FIGURE 7:**
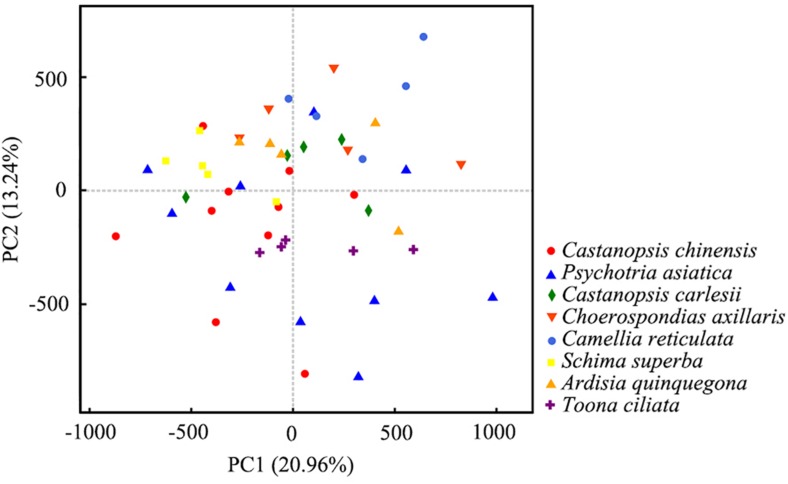
Principle component analysis (PCA) plot of the host plant and soil bacterial communities’ richness. The values of PC1 and PC2, explaining 20.96 and 13.24% of the variance.

To explore the effect of host plants on soil bacterial, we analyzed the mycosphere bacterial communities of *R. griseocarnosa* under different host plants by PCA. The first two axes of the PCA explained 20.96 and 13.24% of the variance in the OTU data, respectively. PCA showed that the samples were dispersed among different host plants (Figure 7). It indicates that the host plant had little effect on soil mycosphere bacteria. There were no significant differences in the bacterial diversity index among the five replicates in each square (data not shown), which indicates that the host plant individual has a minimal effect on bacterial diversity.

#### Environmental Factors Influence the Mycosphere’s Soil Bacteria Communities

The diversity index was significantly correlated with soil and site properties (Table 3). The OTUs and phylogenetic diversity had a positive correlation with geological location altitude, SOC, and AN (Table 3). The Shannon index was significantly and positively correlated with SOC (*p* = 0.012) and AN (*p* = 0.006), while negatively correlated with pH (*p* = 0.012) (Table 3). Collection mycosphere sites had an acidic soil with sample pH values ranging from 3.99 to 4.55.

**TABLE 3:** The Spearman correlation matrix between soil/site properties and diversity indexes.

		Altitude	pH	SOC	AN	AP	AK	OTU	Chao	Shannon	Coverage
pH	*r*	−0.327*									
	*p*	0.02									
SOC	*r*	0.622**	−0.343*								
	*p*	0	0.015								
AN	*r*	0.701**	−0.479**	0.811**							
	*p*	0	0	0							
AP	*r*	0.082	−0.323*	0.572**	0.530**						
	*p*	0.57	0.022	0	0						
AK	*r*	–0.067	−0.325*	0.251	0.439**	0.630**					
	*p*	0.646	0.021	0.078	0.001	0					
OTU	*r*	0.298*	–0.214	0.295*	0.335*	0.087	0.253				
	*p*	0.036	0.136	0.037	0.017	0.547	0.077				
Chao	*r*	0.186	–0.083	0.142	0.14	–0.026	0.135	0.869**			
	*p*	0.195	0.566	0.325	0.332	0.857	0.349	0			
Shannon	*r*	0.259	−0.353*	0.352*	0.382**	0.222	0.252	0.807**	0.557**		
	*p*	0.069	0.012	0.012	0.006	0.122	0.077	0	0		
Coverage	*r*	–0.059	–0.019	–0.007	0.007	0.115	–0.107	−0.755**	−0.939**	−0.368**	
	*p*	0.685	0.896	0.963	0.962	0.426	0.461	0	0	0.009	
PD	*r*	0.399**	–0.172	0.335*	0.337*	0.001	0.134	0.946**	0.888**	0.677**	−0.795**
	*p*	0.004	0.233	0.017	0.017	0.992	0.353	0	0	0	0

r represents the Spearman’s correlation coefficient. SOC, AN, AP, and AK represent soil organic carbon, available nitrogen, available phosphorus, and available potassium, respectively. PD represents phylogenetic diversity. Significant differences by *p < 0.05 and **p < 0.01.

**FIGURE 8:**
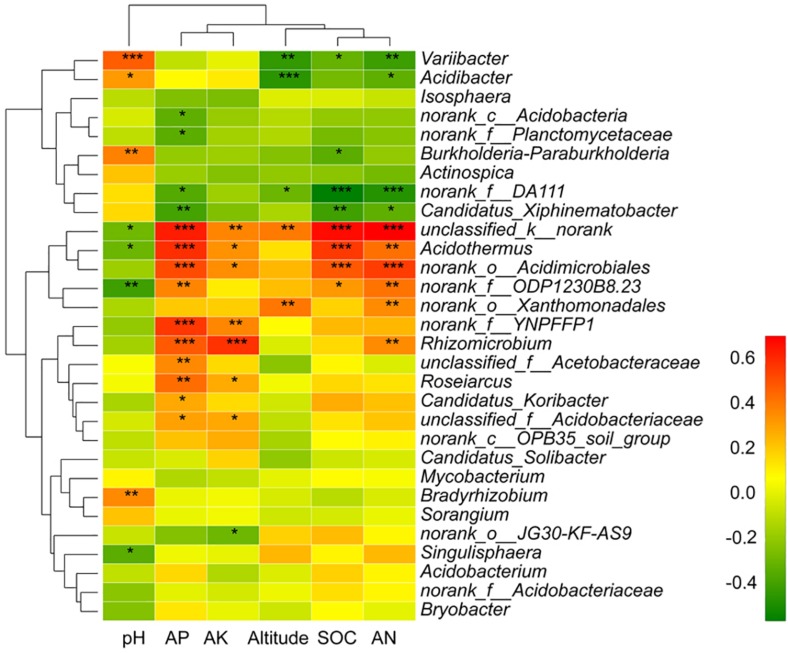
The Spearman correlation of the top 30 genera and soil/site properties. Significant differences by *p < 0.05; **p < 0.01 and ***p < 0.001.

The relative abundance of the top 30 genera and soil/site properties was examined by Spearman correlation analysis (Figure 8). The heatmap showed that AP and AK clustered together and altitude, SOC, and AN clustered together, while pH was further apart on the ordination (Figure 8). *Variibacter* showed a significant positive correlation with pH (*p* < 0.001) and a significant negative correlation with altitude (*p* = 0.002), SOC (*p* = 0.029), and AN (*p* = 0.003). *Acidibacter* showed a negative correlation with altitude (*p* < 0.001) and AN (*p* = 0.021). *Burkholderia-Paraburkholderia* showed a significant positive correlation with pH (*p* = 0.005) and a significant negative correlation with SOC (*p* = 0.018). *Candidatus_Xiphinematobacter* presented a negative correlation with AP (*p* = 0.005), SOC (*p* = 0.004), and AN (*p* = 0.021). *Acidothermus* showed a significant positive correlation with AP (*p* < 0.001), AK (*p* = 0.015), SOC (*p* < 0.001), and AN (*p* = 0.002) and a significant negative correlation with pH (*p* = 0.042). *Rhizomicrobium* showed positive correlation with AP (*p* < 0.001), AK (*p* < 0.001), and AN (*p* = 0.010). *Roseiarcus* showed a positive correlation with AP (*p* = 0.001) and AK (*p* = 0.049). *Candidatus_Koribacter* showed a significant positive correlation with AP (*p* = 0.043). *Bradyrhizobium* showed a significant positive correlation with pH (*p* = 0.0093). *Singulisphaera* showed a significant negative correlation with pH (*p* = 0.017) (Figure 8).

### Functional Predicted in Mycosphere and Bulk Soil

**FIGURE 9:**
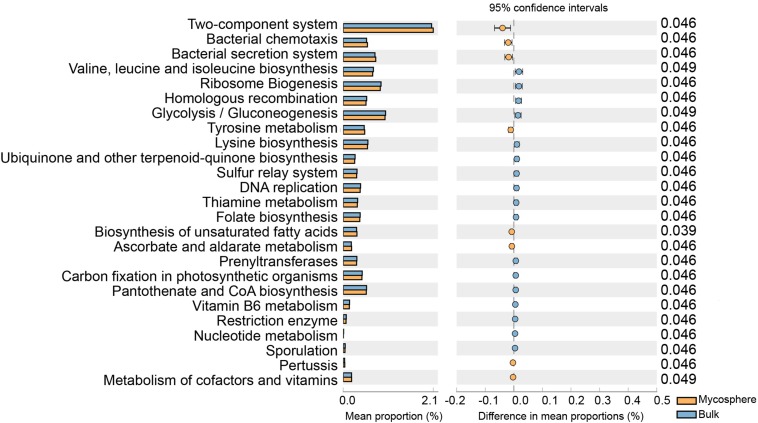
Comparison of the Kyoto Encyclopedia of Genes and Genomes function between mycosphere and bulk soil.

Using the Kyoto Encyclopedia of Genes and Genomes ortholog pathways (Oh et al., 2016), the KEGG functions of the identified bacteria were determined to be significantly (*p* < 0.05) affected by the mycosphere and bulk soil (Figure 9). The results showed that some functional traits, such as two-component system, bacterial chemotaxis, bacterial secretion system, tyrosine metabolism, biosynthesis of unsaturated fatty acids, ascorbate and aldarate metabolism, and metabolism of cofactors and vitamins, were significantly increase in mycosphere soil (*p* < 0.05) (Figure 9). When compared with bulk soil, valine, leucine, and isoleucine biosynthesis, ribosome biogenesis, homologous recombination, glycolysis/gluconeogenesis, and lysine biosynthesis were significantly (*p* < 0.05) lower in mycosphere soil (Figure 9).

## Discussion

### Keystone Species and Ecological Functions

A considerable proportion (∼96%) of the coverage sequences is annotated to bacterial members (Figure 1), indicating that sequencing can be used to analyze the changes of the bacterial community structure in soil samples. Consistent with most of the earlier fungi research (Gryndler et al., 2000; Warmink and van Elsas, 2008; Oh et al., 2016), we found that, for most sites, bacterial diversity in the mycosphere soil was significantly lower than that in bulk soil. As seen in the *R. griseocarnosa* mycosphere soil (Figure 2), low bacterial diversity may be a common feature of the environment in which mycelium dominates (Gryndler et al., 2000). Compared to the bulk soil, *Laccaria* mycosphere bacterial diversity was significantly (*p* < 0.05) reduced on R2A agar analyses (Warmink and van Elsas, 2008). The bacterial diversity *of Tricholoma matsutake* dominant soil was significantly (*p* < 0.05) lower than *T. matsutake* minor soil (Oh et al., 2016). Olsson et al. (1996) demonstrated that ectomycorrhizal hyphae decreased the activity of bacteria in the soil. Therefore, it suggests that the variation of bacterial diversity might reflect the change of *R. griseocarnosa* population.

*Proteobacteria*, *Acidobacteria*, *Actinobacteria*, and *Chloroflexi* were the dominant bacterial communities in the soil (Figure 3), with an overall relative abundance higher than 86%. *Proteobacteria*, *Planctomycetes*, and *Verrucomicrobia* were significantly higher in the mycosphere soil, while *Chloroflexi*, *Firmicutes*, *Cyanobacteria*, *Saccharibacteria*, and *Gemmatimonadetes* were significantly lower. In some soil samples, the content of *Acidobacteria* in mycosphere soil was significantly (*p* < 0.05) higher than that in bulk soil (Supplementary Table S3).

*Proteobacteria* are naturally abundant in soil environments; thus, the increased richness found in the mycosphere soil could be the result of a positive effect of *R. griseocarnosa* because of its fast growth rate and its ability to use the major of root carbon substrates (Lauber et al., 2009). *Proteobacteria* increased richness might be stimulated by the higher nutritional status of soil in the mycosphere (Torsvik and Øvreås, 2002). Moreover, the dominance of *Proteobacteria* in hyphae (Cho et al., 2003), fruit bodies (Barbieri et al., 2010; Pent et al., 2017), and mycorrhizal roots (Poole et al., 2001; Frey-Klett et al., 2007) may be a result of the increased carbon content of these fungal-growing soils. Burke et al. (2006) described *Acidobacterium* as a MHB. Studies have shown that these *Proteobacteria* and *Acidobacteria* are physiologically and ecologically close, and both favor similar ecological niches in the rhizosphere soil (Singh et al., 2007; Kielak et al., 2016). *Planctomycetes* and *Verrucomicrobia* were significantly higher in plant rhizosphere soil (Stafford et al., 2005; Zul et al., 2007; Nunes da Rocha et al., 2009), and they seem to have a strong rhizospheric capacity functionally, but their role in the rhizospheric process remains to be proven.

Bacterial communities displayed distinct structures in the mycosphere and bulk soils (Figure 4 and Supplementary Table S4). *Burkholderia-Paraburkholderia*, *Mycobacterium*, *Roseiarcus*, *Candidatus_Xiphinematobacter*, *Sorangium*, *Acidobacterium*, and *Singulisphaera* were more abundant in the mycosphere soil than in the bulk soil samples (Figure 4 and Supplementary Table S4). The *Proteobacteria* genera *Bradyrhizobium*, *Burkholderia-Paraburkholderia*, and *Roseiarcus* are found in fungi-associated bacterial communities (Pent et al., 2017). For example, *Burkholderia* (Nguyen and Bruns, 2015) is known to be a mycorrhiza helper bacterium that promotes the growth and colonization of mycorrhizae. Kataoka et al. (2008) demonstrated that *Burkholderia* spp. and *Bradyrhizobium* spp. from ectomycorrhizal short roots with *Russula* and *Suillus*. *Burkholderia* spp. are well known as nitrogen-fixing bacteria (Timonen and Hurek, 2006). In recent years, many *Burkholderia* were reclassified as *Paraburkholderia* or *Caballeronia* (Sawana et al., 2014). For example, *Burkholderia phenazinium* and *Burkholderia sordidicola* were moved to the genus *Paraburkholderia* (Sawana et al., 2014), which are found in the mycorrhizosphere of *Pinus muricata* (Nguyen and Bruns, 2015). There is evidence that *Burkholderia* preferentially associates with mycorrhizal and that its strains can spread to the root tip (Poole et al., 2001). The members of the genus *Burkholderia* occur simultaneously with fungal taxa (Stopnisek et al., 2015), and the co-occurring might be due to *Burkholderia*’s ability to migrate with the growing hyphae (Nazir et al., 2012). *Mycobacterium* has nitrogen fixation functions (Rilling et al., 2018) and can provide nitrogen for the growth of *R. griseocarnosa*. *Sorangium* has rich xylan-degrading enzymes that can degrade biological macromolecules, cellulose, hemicellulose, and xylan (Tamaru et al., 2010), which is beneficial for increased mushroom productivity (Zhou et al., 2017). *Singulisphaera*, as an acidophilus, is also found in the rhizosphere soil of *Boletus edulis* (Mediavilla et al., 2019). *Acidobacterium* was significantly higher in plant rhizosphere soil (Oh et al., 2012; Yang et al., 2012), but their role remains to be proven in the rhizospheric process. It is indicated that *Burkholderia-Paraburkholderia*, *Mycobacterium*, *Roseiarcus*, *Acidobacterium*, *Sorangium*, and *Singulisphaera* were MHB of *R. griseocarnosa*. Although the functions of *Candidatus Xiphinematobacter* are unknown, it is possible that *Candidatus Xiphinematobacter* may be a MHB of *R. griseocarnosa*. These bacteria may play important roles in the growth of *R. griseocarnosa*.

### Determinants of Bacterial Communities in Soil

The growth environment of the mycelium (ectomycorrhizal and mycosphere) affects both biological and abiotic factors in the soil ecosystem (Boersma et al., 2010; Kluber et al., 2010; Trappe et al., 2012). Through the study of fungi and bacteria in the mycosphere soil of *T. matsutake*, the results showed that the microbial diversity, community structure, and bacterial function in different geographical locations were similar (Oh et al., 2016). The diversity and community structure of mycosphere soil bacteria of *Agaricus sinodeliciosus* were different in different regions, but they all contained several main taxa (Zhou et al., 2017). *R. griseocarnosa* can co-exist with host tree species such as *Betulaceae*, *Fagaceae*, *Pinaceae*, and *Tiliaceae* to form ectomycorrhiza (Yu et al., 2020), but the symbiosis mechanism is still unclear (Yu et al., 2020), so we mainly studied the relationship between *R. griseocarnosa* and soil bacteria. There is growing evidence that root secretions regulate the relationship between mushrooms and soil microorganisms (Poole et al., 2001; Oh et al., 2016; Pent et al., 2017).

*Russula griseocarnosa* mycosphere has a high AN content in mycosphere soil (Table 1). Increased nitrogen supply can stimulate *Russula* to produce more spores and colonize more oak seedling roots (Avis et al., 2003). Soil pH and AN were significantly higher than most of the mycosphere soil samples (Supplementary Table S2). It was inferred that the main impact factors of *R. griseocarnosa* growth were pH and AN; moreover, previous research has found that pH significantly affects the soil’s bacterial community diversity (Fierer and Jackson, 2006; Rousk et al., 2010; Pent et al., 2017). Singh et al. (2008) found that fungal mycorrhizosphere and bacterial assemblage were affected by the soil pH. Here, the selected study locations had an acidic soil with pH values ranging from 3.99 to 4.55. Previous research showed that the changes in soil microbial community structures were closely related to soil chemistry (Cao et al., 2016). Several soil characteristics (e.g., nutrient availability and organic carbon) are directly or indirectly associated with soil pH, which may contribute to changes in the bacterial community structure (Rousk et al., 2010). Studies have found that higher (Singh et al., 2014) and medium (Meng et al., 2012; Siles and Margesin, 2016) elevations increase bacterial diversity, which is consistent with our findings that medium elevations increase bacterial diversity. The host plants and plant individuals have less of an effect on the diversity of soil rhizosphere bacteria, which is consistent with a previous study (Pivato et al., 2009).

### Bacterial Function

Our study analyzed whether the bacterial communities of the mycosphere and bulk soils produce distinct functional profiles, thus linking *R. griseocarnosa* to specific functions of the bacterial soil. Our results indicated that mycospheres and bulk soils were functionally distinct. Mycosphere soils had an increase in the two-component system, bacterial chemotaxis, bacterial secretion system, tyrosine metabolism, biosynthesis of unsaturated fatty acids, ascorbate and aldarate metabolism, and metabolism of cofactors and vitamins (*p* < 0.05) (Figure 9). *Pseudomonas* can promote the growth of *Agaricus bisporus*, and the autophagy compounds secreted by *A. bisporus* can be degraded by *Pseudomonas* (Chen et al., 2013). Root exudates contain carbohydrates, amino acids, fatty acids, and vitamins, serve as a substrate for mycosphere microorganisms, and provide an important carbon source for soil microbes, thus contributing to the enrichment of the soil microbial community (Bais et al., 2006; Michielse et al., 2012). The increase of nutritional metabolism indicates that these bacteria prefer *R. griseocarnosa* mycosphere soil because it is easier to acquire nutrients (Oh et al., 2016). Although there are limitations in the interpretation of functional predictions, we have identified functions that have potentially positive impacts on *R. griseocarnosa*. Future research can address these functions to elucidate the dynamics among microorganisms in the *R. griseocarnosa* mycosphere soil.

The core functional genes in the mycosphere are not limited to a specific taxon (Yan et al., 2017). The relative abundance of some functional genes in the mycosphere was higher than in bulk soil, indicating that these functional traits were selected by the mycosphere. Although the mechanisms for the functional selection and its consequences in the mycosphere are unclear, our study provides valuable information to better understand the overly complex process of microbial community combinations in the mycosphere soil.

## Conclusion

In conclusion, we identified a suitable environment for *R. griseocarnosa* growth by comparing the physicochemical properties, bacterial diversity, and community structure of mycosphere and bulk soils. 16S rRNA sequencing showed that the bacterial community composition in the mycosphere was significantly different from that of bulk soils. Further analysis showed that *R. griseocarnosa* growth caused a change in the microbial community structure. Growth of *R. griseocarnosa* reduces the diversity and abundance of soil bacterial communities. Among the soil variables, altitude and pH displayed significant contributions in bacterial community structure and diversity properties in all geographical sites under study. Soil pH and AN were the main factors contributing to *R. griseocarnosa* growth. We identified several dominant bacteria genera, including *Mycobacterium*, *Roseiarcus*, *Candidatus_Xiphinematobacter*, *Sorangium*, *Acidobacterium*, and *Singulisphaera* in the mycosphere that may improve *R. griseocarnosa* growth. In the functional analysis, we identified functional modules related to bacterial nutrient metabolism in the *R. griseocarnosa* mycosphere soil. The mycosphere soil is a complex environment, and our study shows that multiple symbiotic relationships between microbes and *R. griseocarnosa* might decrease bacterial diversity. Moreover, it suggests that the fruiting body formation of *R. griseocarnosa* may be affected not only by the host plants but also by the bacterial community in the mycosphere soil. Therefore, the application of management measures to improve soil properties, including the use of N fertilizer and microbial fertilizer containing MHB, may promote the conservation, propagation, and sustainable utilization of *R. griseocarnosa*.

## Data Availability Statement

The Illumina sequencing raw reads were deposited into the NCBI BioProject: PRJNA553654 within GenBank. The SRA accession of raw reads number is SUB5929895.

## Author Contributions

FY, J-FL, and JS participated in study design, sample collection, and statistical analyses. J-KL and S-KW conducted molecular biology experiments. FY drafted the manuscript. J-FL improved the manuscript.

## Conflict of Interest

The authors declare that the research was conducted in the absence of any commercial or financial relationships that could be construed as a potential conflict of interest.

## Footnotes

1 http://drive5.com/uparse/

2 www.i-sanger.com
